# Mucormycosis Caused by *Apophysomyces elegans*—A Case Report and Systematic Review of the Literature of Rhino-Orbito-Cerebral Cases of the Genus *Apophysomyces*

**DOI:** 10.3390/jof11050368

**Published:** 2025-05-09

**Authors:** Vincent Landré, Felix Karl-Ludwig Klingebiel, Christiaan Hendrik Bas van Niftrik, Elisabeth Goetze, Roberto F. Speck, Christian Thomas Hübner, Hans-Christoph Pape, Frank Peter Schäfer

**Affiliations:** 1 Department of Traumatology, University Hospital Zurich, 8091 Zurich, Switzerland; felixkarl-ludwig.klingebiel@usz.ch (F.K.-L.K.); christianthomas.huebner@usz.ch (C.T.H.); hans-christoph.pape@usz.ch (H.-C.P.); frankpeter.schaefer@usz.ch (F.P.S.); 2 Department of Neurosurgery, University Hospital Zurich, 8091 Zurich, Switzerland; bas.vanniftrik@usz.ch; 3 Department of Cranio-Maxillo-Facial and Oral Surgery, University Hospital Zurich, 8091 Zurich, Switzerland; elisabeth.goetze@usz.ch; 4 Department of Infectious Diseases and Hospital Epidemiology, University Hospital Zurich, 8091 Zurich, Switzerland; roberto.speck@usz.ch

**Keywords:** mucormycosis, *Apophysomyces*, *Apophysomyces elegans*, polytrauma, HIV

## Abstract

Introduction: Orbitocerebral mucormycosis, caused by *Apophysomyces*, is a rare infection, usually occurring in tropical and subtropical climates, with a high mortality rate. We report a case of orbitocerebral mucormycosis caused by *A. elegans* in a person living with HIV (PLWHIV) from Africa alongside a systematic literature review updating current diagnostic and treatment strategies for orbitocerebral mucormycosis caused by *Apophysomyces*. Methods: The presented case was treated in our hospital for polytrauma following a motor vehicle accident (MVA) with aggressive surgical debridement and therapy with liposomal Amphotericin B (AMB). We evaluated clinical presentation, imaging, surgery, and postoperative outcomes. A systematic review of English or German language articles (published between 1985 and 2025) was performed according to PRISMA guidelines. Articles describing patients with mucormycosis due to *Apophysomyces* were summarized. Quantitative values for relevant parameters that indicated a reduction in mortality and morbidity were obtained. Results: The systematic search initially identified 452 publications, from which 79 studies were retrieved. Seventeen publications comprising 21 cases were included, along with one additional case from our institution, for a total of 22 rhino-orbito-cerebral infections caused by the genus *Apophysomyces*. *Apophysomyces elegans* (*A. elegans*) was the most frequently isolated species (n = 17), followed by *A. variabilis* (n = 4) and *A. ossiformis* (n = 1); *A. trapeziformis* was not reported. The majority of patients were male (72.7%), with a mean age of 40.7 ± 15.9 years. Trauma (27.3%) and diabetes mellitus (18.2%) were the most common underlying risk factors, with SARS-CoV-2 infection identified in 13.6% of cases. Conclusion: Mucormycosis due to *Apophysomyces* is a rare but potentially devastating condition. Based on our experience and the literature, we suggest that the early diagnosis of *Apophysomyces* treated with liposomal AMB and aggressive surgical debridement is essential to reduce morbidity and mortality.

## 1. Introduction

*Apophysomyces*, which belongs to the order Mucorales, has gained recognition in recent decades due to its potential to cause severe, often fatal infections in immunocompetent individuals [1]. Recent reviews highlight an increasing incidence of trauma-related mucormycosis, particularly after motor vehicle accidents and natural disasters [2,3]. *A. elegans* was the first *Apophysomyces* discovered and was initially described by Misra in 1979; the taxonomy and clinical significance have been recognized with increasing reports of infections worldwide, particularly from tropical and subtropical regions [4,5]. The clinical spectrum of *Apophysomyces* infections includes diverse manifestations ranging from localized cutaneous infections to aggressive disseminated disease involving vital organs. Cutaneous forms often result from traumatic injury, surgical wounds, or burns where the fungus invades and multiplies in the subcutaneous tissue. However, its ability to rapidly progress to invasive disease involving deeper structures and organs poses a significant challenge to clinical management [6]. Therapeutically, the management of *Apophysomyces* infections remains complex. Surgical debridement combined with systemic antifungal therapy is the mainstay of treatment [7]. However, the limited repertoire of antifungal agents effective against *A. elegans*, coupled with the emergence of resistance, underscores the urgent need for novel therapeutic strategies and a deeper understanding of their virulence mechanisms. This review aims to provide a comprehensive overview of *Apophysomyces*, including their epidemiology, pathogenesis, clinical manifestations, diagnostic challenges, and therapeutic interventions. By synthesizing existing knowledge and highlighting research gaps, this manuscript aims to contribute to a better understanding of this clinically important fungal pathogen and guide future research efforts toward improved patient outcomes.

## 2. Case Report

**Figure 1:**
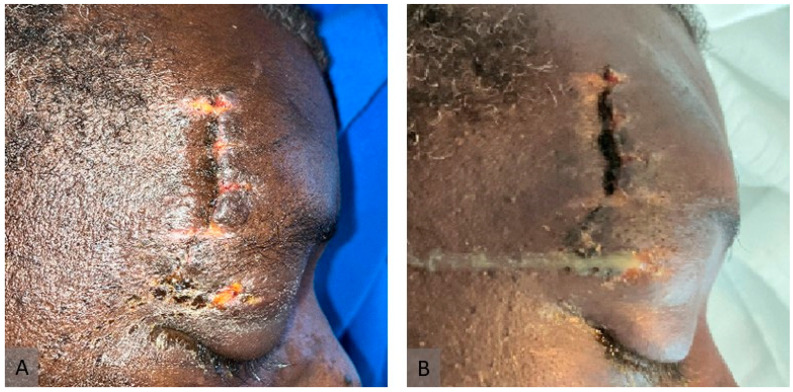
**Clinical presentation of murocormycosis.** (**A**) One day before the onset of symptoms (day 11 of hospitalization). (**B**) Immediately after the onset of symptoms (day 12 of hospitalization).

**Figure 2:**
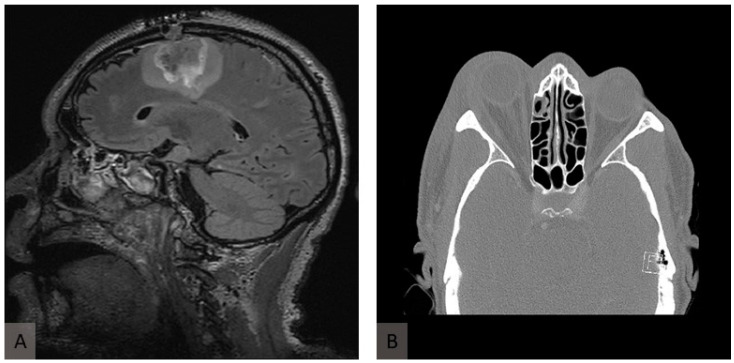
**Radiological presentation of murocormycosis.** (**A**) Axial fluid-attenuated inversion recovery (FLAIR) image of T2-weighted MRI of the head showing inflammatory changes in the frontal and parietal lobes in the sagittal plane. (**B**) A computed tomography scan at the initial presentation shows proptosis of the right eye and retrobulbar inflammation.

We present the case of a 54-year-old male person living with HIV (PLWHIV) (HIV-1, CDC stage B3) who was involved in a motor vehicle accident (MVA) in Nigeria. HIV infection had been diagnosed in 1997, 26 years prior to the diagnosis of mucormycosis. From the initial diagnosis, the patient was on continuous antiretroviral therapy, currently consisting of daily Emtricitabine (50 mg), Tenofovir alafenamide (200 mg), and Bictegravir (25 mg). At the time of mucormycosis diagnosis, the patient’s HIV-1 RNA was suppressed to <20 copies/mL, and the CD4+ lymphocyte count was 528 cells/μL. The patient was initially treated in a local hospital for four days, where only contused lacerations on the right side of the face were treated, and no further assessment was undertaken. The patient then flew commercially to Switzerland for further treatment at the University Hospital of Zurich. Upon admission, the patient fulfilled the criteria for polytrauma, presenting multiple severe injuries. The cranial assessment revealed a concussion accompanied by a right monocular hematoma. Thoracic trauma included a multifragmentary, dislocated clavicular shaft fracture; a multifragmentary, dislocated scapular fracture; and severely dislocated fractures of the first to ninth ribs, resulting in a flail chest. These symptoms were further complicated by pneumothorax and marked pneumomediastinum. Spinal injuries comprised slightly displaced fractures of the transverse processes of the first and second lumbar vertebrae. Additionally, a tibial head fracture was identified and managed via osteosynthesis. On arrival, no evidence of mucormycosis was noted. On day 12 of hospitalization, the patient developed focal signs of infection and pus, initially interpreted as a bacterial infection over the right eye (Figure 1A,B). Initially, neurological involvement of the infection could not be ruled out, as MRI was contraindicated due to possible residual metal from the accident in the skull and the onset of delirium, coupled with the patient’s refusal of debridement due to potential morbidity on day 13 of hospitalization. Therefore, only broad-spectrum antibiotic therapy with Piperacillin/tazobactam 4.5 g every 8 h could be initiated. After obtaining the patient’s consent, debridement of the infected area was performed, with the subsequent submission of the debrided tissue for culture and histological analysis, which presented Staphylococcus epidermidis. Despite broad-spectrum antibiotics, surgical debridement by a multidisciplinary team, and vigilant wound care, the patient’s condition deteriorated. Forty days after the accident and 28 days after the onset of symptoms, *A. elegans* was identified by sequencing 18S rRNA from soft tissue. Homology with the GenBank 18S rRNA sequence showed 0 mismatches and 100% identity. Primers for identification were used according to the broad-coverage fungal quantitative real-time PCR assay FungiQuant [8]. A microbiological report is attached to this study. The most likely route of transmission of *A. elegans* was the contamination of wounds at the accident site. However, infection from contaminated surgical sutures or postoperative dressings during the initial treatment in Nigeria could not be excluded. Immediately after the identification of *A. elegans* as the cause of mucormycosis, treatment with liposomal Amphotericin B (Ambisome) at 10 mg/kg body weight i.v. every 24 h, and Isavuconazole at 200 mg every 8 h was initiated. In addition, local irrigation of the wound with AMB and aggressive surgical debridement were continued. As emphasized by Cornely et al. (2019), early surgical debridement remains the cornerstone of successful management in cutaneous mucormycosis [2]. Due to the rhino-orbito-cerebral nature of the infection, the patient underwent right orbital exenteration. Neurological involvement of the infection (Figure 2A) was discovered after an MRI could be performed, and multiple cerebral abscess evacuations were subsequently carried out by neurosurgeons. The hospitalization was complicated by pneumonia, pleocytosis, cerebral venous sinus thrombosis (treated from day 20 of hospitalization with low-molecular-weight heparin), acute myocardial injury, and delirium. The disease was controlled by multiple debridements performed by different specialties, orbital enucleation, and liposomal AMB treatment. The patient was initially discharged for rehabilitation 116 days after admission following successful infection control. After rehabilitation, the patient was readmitted twice for mucormycosis recurrence and once for pneumonitis. Antifungal and antibiotic therapy were restarted, and further debridements were performed. In total, the patient spent 259 days in hospital or rehabilitation. Unfortunately, the patient died due to sepsis of an unknown origin, leading to rapid deterioration with respiratory insufficiency, lactate increase, and hypoglycemia. It became clear that stabilization would not be possible without intensive care for multiple organ failure. After interdisciplinary discussion and conversations with the patient’s family, treatment was switched to the best supportive care due to exhausted infectious and surgical treatment options and the overall poor prognosis.

## 3. Material and Methods

Following the PRISMA guidelines, a systematic review of peer-reviewed articles in the English and German languages published between 1985 and 2025 was performed. If a study was human-related, related to rhino-orbito-cerebral infections due to the genus *Apophysomyces*, published in the given period and reported in English or German, it was eligible and included in this research project. Exclusion criteria were books, correspondence, conference abstracts, expert opinions, editorials, and in vitro/vivo experiments. The primary sources of information were PubMed^®^ and EMBASE^®^. Cases that were part of reviews without focusing on *Apophysomyces* and detailed case descriptions were excluded from our systematic review [3]. The search was conducted in March 2025. For the literature research, we used “apophysomyces” as a search term in both full-text archives and received 176 results in PubMed^®^ and 276 results from EMBASE^®^. After the removal of duplicates, we conducted a full-text review of 303 publications and identified 22 cases, including our case of rhino-orbito-cerebral infections due to the genus *Apophysomyces*. For study selection, two authors screened articles in a blinded fashion, and disagreements were resolved by consensus. The included articles were then screened for relevant demographics, risk factors, treatment, and outcomes. Due to the small number of cases, no statistical techniques were used to analyze the data.

## 4. Results

**Figure 3:**
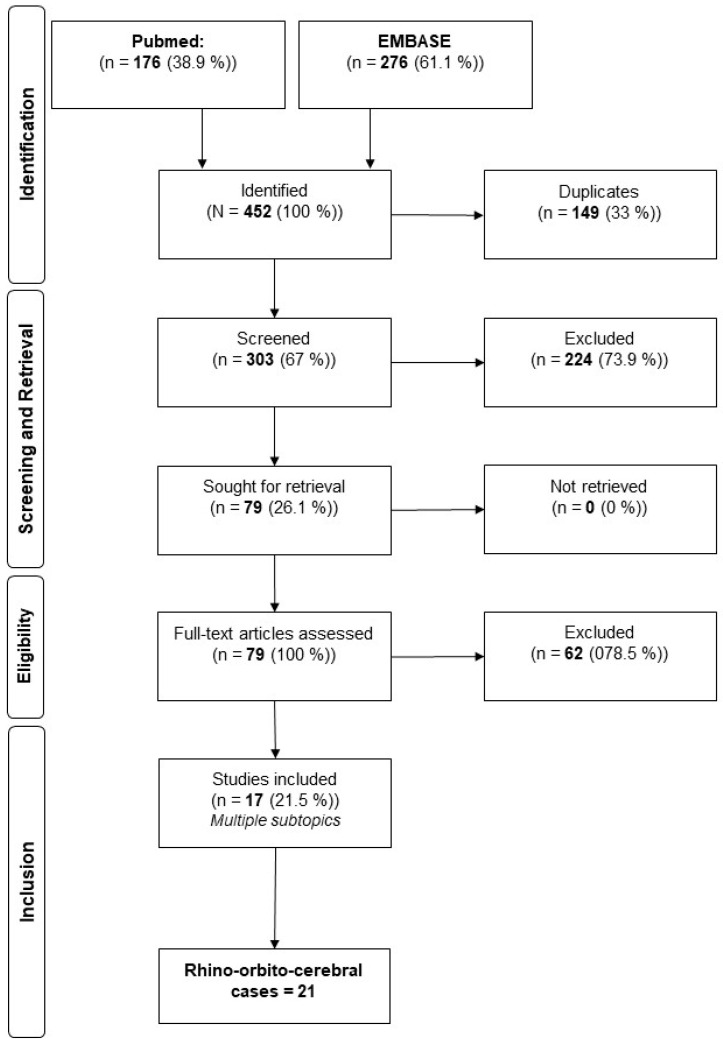
Flowchart of the literature analysis.

The initial systematic search using Medical Subject Headings criteria yielded 452 publications considered relevant to rhino-orbito-cerebral infections due to the genus *Apophysomyces*. After applying the inclusion criteria, 79 studies were identified for retrieval. Of these, we identified 17 publications that included 21 relevant cases (22 including our own case), and that focused on rhino-orbito-cerebral cases of the genus *Apophysomyces* (Figure 3). Quantitative values for relevant parameters that indicated a reduction in mortality and morbidity were obtained. The treatment of mucormycosis due to *Apophysomyces* and a general overview is given in Table 1. The review of reported cases of rhino-orbito-cerebral infections caused by *Apophysomyces* species shows a clear predominance of *Apophysomyces elegans*. Out of all documented cases, *A. elegans* was responsible for 17 infections, making it the most frequently isolated species in this clinical setting. *Apophysomyces variabilis* accounted for four cases, indicating that it plays a lesser but still notable role in such infections. *Apophysomyces ossiformis* was associated with only a single case, highlighting its rarity as a cause of rhino-orbito-cerebral mucormycosis. *Apophysomyces trapeziformis* was not implicated in any reported cases of this type of infection. A total of 22 cases of rhino-orbito-cerebral infections caused by the genus *Apophysomyces* were analyzed (Table 1). The majority of the patients were male (n = 16, 72.7%), while females accounted for 27.3% of cases (n = 6). The mean age of the cohort was 40.7 years, with a standard deviation (SD) of 15.9 years. Regarding underlying risk factors, trauma was the most common, present in 27.3% of cases (n = 6), followed by diabetes mellitus in 18.2% (n = 4). SARS-CoV-2 infection was identified as a risk factor in 13.6% of cases (n = 3). Less common predisposed conditions included myelofibrosis and HIV infection, each observed in 4.5% of patients (n = 1, respectively).

## 5. Discussion

*Apophysomyces* is a genus of thermotolerant fungi within the order *Mucorales* and family *Mucoraceae*, known for causing severe infections, predominantly in immunocompetent hosts after traumatic injuries. Species within this genus include *Apophysomyces elegans*, *Apophysomyces variabilis*, *Apophysomyces ossiformis*, *Apophysomyces trapeziformis*, and *Apophysomyces mexicanus*. These fungi are found primarily in soil and decaying vegetation in tropical and subtropical climates. The genus as a whole is unique among *Mucorales* because it predominantly infects immunocompetent individuals following trauma rather than immunocompromised hosts [25]. The first species, *A. elegans*, was discovered in a mango orchard in northern India [5]. While other species like *A. variabilis* and *A. trapeziformis* have been isolated in different regions, *A. elegans* remains the most commonly reported species. Compared to other zygomycotic infections, the cases caused by *Apophysomyces* species may have lower mortality because most infections occur in previously healthy individuals who have suffered a traumatic event [3,26]. Since the early 21st century, infections caused by *Apophysomyces* species have become increasingly recognized, often presenting with high morbidity and mortality, particularly following traumatic events [20]. The clinical manifestation of mucormycosis caused by *Apophysomyces* varies, depending on the patient’s predisposing factors and the portal of entry [27]. The five main clinical forms are gastrointestinal, rhino-orbito-cerebral, pulmonary, primary cutaneous, and disseminated mucormycosis [6]. As with other mucormycoses, typical pathological features include angioinvasion, thrombosis, and necrosis [14]. The present case also showed typical cerebral venous sinus thrombosis in the infected region. In this article, we focus primarily on the rhino-orbito-cerebral route of infection due to the case presented. With the inclusion of our case, we found 22 cases of rhino-orbitocerebral *Apophysomyces* in the literature. We present the first case in the literature of *A. elegans* acquired on the African continent. Patients with *A. elegans* infection have been documented in India, the southern United States, Australia, Mexico, the Caribbean Islands, Colombia, and Venezuela. However, of the nearly 100 cases published in the literature, the majority (60%) are from India [28]. Orbital fungal infections are rare and usually occur in patients with comorbidities such as neutropenia, chronic illness, diabetes, or immunocompromised patients [20]. Uncontrolled diabetes mellitus, particularly when complicated by diabetic ketoacidosis, can be a significant risk factor for rhino-orbito-cerebral mucormycosis due to *Apophysomyces* since elevated blood glucose levels and acidic pH in such conditions create an environment conducive to fungal proliferation and impair neutrophil function, diminishing the body’s ability to combat invasive fungal infections [29]. In immunocompetent patients without a history of trauma, rhino-orbito-cerebral infections may result from several etiologies. Bacterial infections, particularly those secondary to sinusitis, can extend contiguously to involve the orbit and intracranial structures. Fungal infections, notably chronic invasive fungal sinusitis caused by *Aspergillus* species, are also recognized in this patient population. Additionally, congenital anomalies such as encephaloceles or meningoencephaloceles can predispose individuals to infection by creating abnormal communications between the sinonasal tract and the intracranial compartment. Iatrogenic causes, including dental procedures and surgical interventions, may serve as portals of entry for pathogens. Odontogenic infections represent another potential source through direct contiguous spread [15]. *A. elegans* is particularly unique within the order Mucorales due to its propensity to cause infection, primarily in the context of traumatic injury or burns and especially in the context of soil exposure [30]. Interestingly, as discussed later, HIV infection does not promote mucormycosis due to *A. elegans*. We highlight that the most common risk factor for infection with *A. elegans* is contamination following trauma (Table 1). The average time between injury and the onset of symptoms is 10 to 20 days [31]. In the presented case, it was comparable to 16 days. Usually, it takes up to 3 days to make a definitive diagnosis. *Apophysomyces* can only be diagnosed with an adequate tissue sample harvested through the debridement of the affected tissue. Further diagnostics with MRIs can help to assess the extension of the infection. However, MRI alone is not sufficient for diagnosis. The time taken to diagnosis in the presented case was prolonged as *A. elegans* could not be isolated from the wound because the specimen needed for the analysis could not be extracted due to the patient’s refusal to undergo tissue debridement. In addition, imaging was significantly hampered by possible residual metal fragments from the initial trauma. *A. elegans* was not initially suspected as the causative agent, as no case of this fungus has been reported in Europe or Nigeria. Novel molecular tools like Fn-HNP-assisted LAMP and multiplex qPCR targeting for the *cotH* gene enable non-invasive, species-specific detection from blood or urine within 45 min, with sensitivities over 83%. These methods outperform traditional diagnostics by providing earlier results than imaging or histopathology. CT and MRI can support clinical suspicion, particularly in pulmonary or rhino-orbital presentations, through characteristic signs such as the reverse halo sign. Combining rapid molecular assays with imaging offers an effective strategy for timely diagnosis in the ICU and treatment of critically ill patients [32,33]. The prevalence and types of physical trauma may vary depending on factors such as geographical location, socio-economic conditions, and cultural norms. Some general trends are that men suffer more from physical trauma because they are more likely to be employed in physically demanding and dangerous occupations such as construction, mining, and certain manufacturing industries. This may contribute to the higher incidence of mucormycosis in men. All patients with rhino-orbitocerebral received treatment with AMB (Table 1). Normally, treatment with liposomal AMB in combination with radical debridement is the treatment of choice for orbitocerebral mucormycosis [7,26]. In the present case, we also used liposomal AMB because the liposomal form is associated with improved recovery from mucormycosis due to less frequent side effects [34,35]. With a combination of surgery and medical treatment, AMB is significantly better than AMB alone [36]. However, the utility of AMB is limited by its frequent adverse effects, particularly nephrotoxicity, which limits the dose that can be safely administered. The use of formulations of AMB combined with lipid structures can alleviate many of these undesirable side effects due to their reduced toxicity [16,37]. It also appears that the liposomal encapsulation of AMB may improve the targeting of fungi, infected organs, and phagocytes during administration [12]. Due to the invasive nature, the orbit and surrounding tissues were involved, and we had to perform an orbital exenteration. This has previously been associated with an increase in survival in patients with fever [30]. With the inclusion of our case, there are four cases in the literature (20%) in which focal treatment with AMB was performed with either irrigation or soaked dressings (Table 1). We used focal irrigation to increase the targeted affection of liposomal AMB, although there is no evidence that this method reduces morbidity. The efficacy of using AMB-soaked dressings within the wound remains uncertain, but the use of this approach does not appear to be associated with any complications and could potentially benefit the patient [37]. Some authors have shown that hyperbaric oxygen therapy may increase patient survival through several mechanisms, such as optimizing the antifungal capacity of neutrophils [30]. Although the role of hyperbaric oxygen is only theoretical, it reduces tissue hypoxia and acidosis, which could consequently reduce vascular invasion [16]. There are no in vitro data to support this theory, and no treatment recommendation can be made based on these anecdotal data [38]. The success rate of treatment with hyperbaric oxygen and focal therapy, therefore, has limited support for use [37]. Other antifungal drugs such as fluconazole, itraconazole, or nystatin were not sufficient to control the fungal infection [7,26]. Broad-spectrum antibiotics may even worsen the prognosis, as they may promote mucormycosis by eliminating bacterial competition [16]. Posaconazole has been shown to be effective in treatment, especially in palliative settings, while *A. elegans* appears to be resistant to other antifungal agents such as itraconazole and voriconazole [28,39]. In the present case, Isavuconazole at 200 mg every 8 h was used to treat the invasive mucormycosis. It is debatable whether this drug helped to control the initial infection or whether this was achieved by the established treatment of liposomal AMB in combination with radical debridement. The role of immunosuppressants and corticosteroids in the treatment of *Apophysomyces* infections is not well-established. While corticosteroids may be used to control inflammation, their potential to exacerbate fungal infections warrants cautious use [40]. Successful treatment requires timely surgical intervention, the systemic administration of antifungal drugs, and addressing the underlying causes of risk. Optimal therapy, therefore, involves a combination of thorough surgical debridement and the administration of AMB at a maximum tolerated dose. The early diagnosis and initiation of appropriate therapy can improve the prognosis for patients with this infection [26,41]. Acquired immune deficiency syndrome (AIDS) is associated with only a few reported infections with *Apophysomyces* and, interestingly, does not appear to be a risk factor for mucormycosis [42]. HIV infection causes a defect in T helper cells. This depletion of T cells alone, or impairment of neutrophil function, does not significantly promote the development of mucormycosis [43]. It is likely that neither T cells nor neutrophils are involved in the defense against mucormycosis [43]. This is underlined by the fact that there is no increased incidence of invasive aspergillosis in HIV patients with drug-induced neutropenia [43]. Although the presented case had a well-controlled HIV infection with the above-mentioned treatment and a questionable compromised immune status, it cannot be concluded that this comorbidity promoted the infection. The prognosis of *Apophysomyces* infection depends on the development of concurrent underlying diseases. Favorable outcomes are less likely in the presence of comorbidities such as diabetes mellitus, granulocytopenia, or other immunocompromised states [26]. Mostly, a cure for mucormycosis due to *A.* *elegans* is associated with high morbidity due to debridement and orbital exenteration. *A. elegans*, like other zygomycetes, invades blood vessels to cause ischemia and tissue necrosis. It is common for facial infarction to be followed by orbital ischemia and further progression into the cranial cavity [30]. In the case presented, cerebral venous sinus thrombosis developed and was treated accordingly with intravenous heparin. It is common for *Apophysomyces* infections to delay the initiation of antifungal therapy due to the long time taken to find a diagnosis. This could worsen the prognosis by delaying the start of appropriate therapy. In the present case, the HIV infection is not expected to have worsened the outcome due to prior adequate therapy. For future therapies, it is expected that local therapy in combination with timely diagnosis could improve the medical outcome. Increasing the average daily dose of AMB has been shown to improve outcomes, although this has not been shown to be statistically significant [37]. Intraocular injections were shown to be effective in palliative cases where aggressive debridement could not be performed [30]. Orbital mucormycosis was also successfully treated with the retrobulbar injection of AMB without exenteration [44]. A multidisciplinary team is required to oversee the management of mucormycosis cases, including extensive surgical debridement, the administration of AMB, and the treatment of underlying predisposing factors [30]. Some authors even discuss that the outcome depends primarily on the prognosis of the underlying disease and early diagnosis and treatment [45]. We assume that factors influencing the outcome depend on the available treatment, early diagnosis, underlying diseases, and the compliance of the patients. Although *Apophysomyces* mucormycosis is rare, its prevalence in the scientific literature may be underestimated due to publication bias and a lack of diagnostic tools in affected areas due to a lack of funding in tropical and subtropical regions. This potential under-representation limits the generalizability of our research findings. The inherent limitations of our retrospective study include variations in the completeness of reported data. To address this methodological concern, our focus was deliberately narrowed to include reported cases with robust and comprehensive data identified through systematic searches of medical literature databases. Other literature has been excluded. It is important to note that at the time of the current literature review, no clinical trials with randomization methods have been documented.

## 6. Conclusions

This study was carried out since *Apophysomyces*, and especially *A. elegans* is a rare pathogen in mucormycosis and the first of its kind to be documented from the African continent. The presence of *Apophysomyces* as the causative agent of mucormycosis warrants consideration in differential diagnosis, especially if the patient has been exposed to tropical or subtropical regions and has a history of trauma. This is true even in the absence of underlying disease and regardless of immunological status. Early recognition and appropriate treatment are essential for the effective management of this potentially life-threatening infection. In line with the literature, we would like to emphasize that treatment with liposomal AMB in combination with aggressive debridement can be successful. As most cases originate from India, we believe that this would be the most feasible location to initiate randomized trials to further explore treatment options with sufficient power.

## Figures and Tables

**Table 1:** Rhino-orbito-cerebral cases of the genus *Apophysomyces*.

Year	First Author et al. (REF)	Age	Sex	Country	Trauma	Symptom Duration Before Presentation	Comorbidities	AMB	Surgical Debridement	Orbital Enucleation	Focal Irrigation	Further Treatment	Outcome
1995	Radner [9]	19	Male	Mexico	Tractor Accident	6 days	Healthy	Yes	Yes	Yes	No	No	Survived
1997	Chakrabarti [10]	52	Male	India	No	5 days	Myelofibrosis	Yes	Yes	Yes	No	No	Died
1997	Sdralis [11]	60	Male	Australia	No	21 days	Healthy	Yes	Yes	Yes	No	Hyperbaric therapy	Survived
2000	Fairley [12]	59	Male	Australia	Eyetrauma	3 days	Healthy	Yes	Yes	Yes	Yes	No	Survived
2001	Garcia-Covarrubias [13]	24	Male	USA	Motorcycle Accident	10 days	Healthy	Yes	Yes	Yes	No	Hyperbaric therapy	Survived
2003	Chakrabarti [4]	20	Female	India	No	7 days	Healthy	Yes	Yes	Yes	No	No	Survived
2006	Liang [14]	50	Male	USA	No	14 days	Diabetic	Yes	Yes	Yes	No	No	Survived
2006	Rao [15]	45	Male	India	No	15 to 28 days	Healthy	Yes	Yes	Yes	No	No	Survived
2006	Rao [15]	26	Female	India	No	15 to 28 days	Healthy	Yes	Yes	Yes	No	No	Survived
2006	Rao [15]	26	Male	India	No	15 to 28 days	Healthy	Yes	Yes	Yes	No	No	Survived
2006	Rao [15]	16	Female	India	No	15 to 28 days	Healthy	Yes	Yes	Yes	No	No	Survived
2006	Rao [15]	55	Male	India	No	15 to 28 days	Healthy	Yes	Yes	Yes	No	No	Died
2007	Schütz [16]	30	Male	India	No	28 days	Healthy	Yes	Yes	Yes	Yes	No	Died
2007	Ferguson [17]	43	Male	USA	Automobile Accident	14 days	Diabetic	Yes	Yes	Yes	Yes	Posaconazole	Survived
2013	Parsi [18]	25	Male	India	Nose Operation	28 days	Healthy	Yes	Yes	No	No	No	Survived
2015	Biswas [19]	45	Female	India	No	Unknown	Diabetic	Yes	No	No	No	No	Survived
2016	Wolkow [20]	74	Female	USA	No	14 days	Healthy	Yes	No	No	No	Isavuconazole	Survived
2020	Martinez-Herrera [21]	46	Male	Mexico	No	Unknown	Diabetic	Yes	No	No	No	No	Died
2022	Erami [22]	40	Female	Iran	No	5 days	SARS-CoV-2	Yes	No	No	No	No	Died
2024	Kaur [23]	32	Male	India	No	2 days	SARS-CoV-2	Yes	Yes	Yes	No	Maxillectomy	Died
2025	Shafran [24]	54	Male	Israel	No	Unknown	Healthy	Yes	No	No	No	Itraconazole	Died
2025	This publication	54	Male	Nigeria	Automobile Accident	4 days	HIV	Yes	Yes	Yes	Yes	Isavuconazole	Died

## Data Availability

All data related to this research are reported in the main text and Supplementary Materials.

## Supplementary Materials

The following supporting information can be downloaded at https://www.mdpi.com/article/10.3390/jof11050368/s1. Microbiological report that revealed *A. elegans* as the cause of infection.
