# Supplementary material for: Mucormycosis Caused by Apophysomyces elegans—A Case Report and Systematic Review of the Literature of Rhino-Orbito-Cerebral Cases of the Genus Apophysomyces
Source: J Fungi (Basel). 2025 May 9;11(5):368. doi: 10.3390/jof11050368 (PMC12112950; doi:10.3390/jof11050368)
Supplement: Supplementary file 1 [file jof-11-00368-s001.zip › jof-3559263-supplementary.pdf]

Universität Zürich[UZH]

Institut für Medizinische Mikrobiologie

Universität Zürich
Institut für Medizinische Mikrobiologie
Gloriastrasse 30 / 32
CH-8006 Zürich
Tel. +41 44 634 27 00
Fax. +41 44 634 49 06
www.imm.uzh.ch

Zürich, 12.12.2023

Universitätsspital Zürich 031
TDR Station SUED B CH2105
Trauma-Derma-Rheuma 4423
Rämistr. 100, SUED B 18
8091 Zürich

Patient: [redacted]
Beh. Arzt:
Auftrag: 50083085

Arbeitsplatz: ngs 2023187374
Material: Gewebe
Folgebericht zum Auftrag Nr. 3'280'892 (v10 2023 865454)

Eingang: 23.06.2023

Molekularbiologischer Endbefund

Next Generation Sequencing **positiv**

Die Identifizierung durch Sequenzierung der 18S-Region ergab: Apophysomyces elegans

Keim	Mismatches	Identität in %
Apophysomyces elegans	0/294	100
Saksenaea vasiformis	14/294	95.2
Backusella variabilis	17/295	94.2

Es hat sich gezeigt, dass die bakterielle Breitspektrum-PCR aus den verschiedensten Gründen weniger sensitiv ist als spezies-spezifische PCR-Systeme. Ein negatives Ergebnis schliesst somit das Vorliegen eines bakteriellen Infektes nicht aus.

Verantwortlich
Freigabe durch: Dr. H. Seth-Smith

Dr. phil. nat. Frank Imkamp, FAMH Mikrobiologie, Tel 103 42667

Hinweis: Zu Angaben betreffend Probenmaterialien, Unterauftragnehmer und Stichprobenrelevanz siehe http://www.imm.uzh.ch. Im Bericht festgehaltene Resultate beziehen sich ausschliesslich auf das untersuchte klinische Material wie erhalten. Meldepflichtige Keime werden automatisch dem BAG/Kantonsarzt weitergeleitet. Der Inhalt des Befundes ist vertraulich und ausschliesslich für den genannten Empfänger bestimmt. Bei irrtümlichem Erhalt dieses Befundes bitten wir um Vernichtung des Inhalts und Information des Absenders. Dieser Befund ist nur gesamthaft gültig und darf nicht auszugsweise kopiert werden, dagegen ist die Weiterverwendung einzelner Resultate mit Angabe der Quelle erlaubt.

Unsere Öffnungszeiten: Montag-Freitag von 8 bis 20 Uhr, Samstag+Sonntag von 8 bis 17 Uhr
